# In-Situ Visualization of the Cell Formation Process of Foamed Polypropylene under Different Foaming Environments

**DOI:** 10.3390/polym13091468

**Published:** 2021-05-01

**Authors:** Rong Deng, Tuanhui Jiang, Chun Zhang, Xiangbu Zeng, Bujin Liu, Jingkui Yang, Shengnan Li, Jun Gu, Wei Gong, Li He

**Affiliations:** 1 College of Materials Science and Metallurgy Engineering, Guizhou University, Guiyang 550025, China; d1142028552@126.com (R.D.); gs.pjliu18@gzu.edu.cn (B.L.); jkyang2006@126.com (J.Y.); 2 National Engineering Research Center for Compounding and Modification of Polymeric Materials, Guiyang 550014, China; jth1983@126.com (T.J.); zxbmold@163.com (X.Z.); lsn_gzyx@163.com (S.L.); 18761200842@163.com (J.G.); 3 School of Materials and Energy Engineering, Guizhou Institute of Technology, Guiyang 550003, China; zhangchun925@126.com; 4 School of Materials and Architectural Engineering of Guizhou Normal University, Guiyang 550025, China

**Keywords:** polypropylene, foaming, dynamic foaming process, cell formation

## Abstract

In this paper, the dynamic foaming process of micro-foaming polypropylene (PP) in different foaming environments in real time was obtained via a visualization device. The relationship curve between cell number (*n*) and foaming time (*t*) was plotted, and then the nucleation kinetics of foam cells was analyzed. Results showed that the formation rate of cells changed obviously with the variation of melt temperature and the content of the foaming agent. The *n*-*t* curves presented a typical “S” shape, which indicated that the appearance of the cell number increased slowly in the initial foaming period, then increased rapidly in a short time, and finally maintained a certain value. When a certain pressure was applied to the PP melt, the external force had a great influence on the *n*-*t* curve. With the increasing external force, the rate of cell formation increased rapidly, and the shape of the *n*-*t* curve changed from “S” to “semi-S” without an obvious slow increase. The investigation of the *n*-*t* relationship in the PP dynamic foaming process under different foaming environments could provide effective bases for improving the foaming quality of injection molding foaming materials.

## 1. Introduction

Microcellular foams possess many excellent properties such as light weight, high strength, good thermal/sound insulation, and high cushioning performance, which enables it to be applied to the materials in packaging, construction, aerospace, automotive decoration, and medical equipment [1,2]. The cell size and distribution directly affect the properties of materials, thus limiting their application in various fields [3]. Therefore, more and more research is devoted to the development of foaming materials with higher cell density and smaller cell size. As known, the formation of the cell structure generally goes through three processes: the formation of cell nucleation, the growth of cell nucleation, and the curing as well as shaping of cells [4,5,6]. Due to the different formation mechanisms at each stage, the main influential parameters are also different. Among them, the cell nucleation stage plays a decisive role in the cell density and distribution of foaming materials. If highly uniform distributed cell nucleation appears in the melt at the same time, high-quality cells with uniform and fine size can be obtained. On the contrary, if the cell nucleation does not occur at the same time in the melt, uneven cell size distribution and small weight loss of the product will be obtained. Therefore, the cell nucleation period is a key stage that affects the performance and quality of the foamed material directly [7,8,9]. The stage of cell growth directly affects the geometry and structure of the cells. The diffusion velocity and penetration velocity of the gas in the melt are important parameters to control the cell growth [10,11]. Whether the final cell can be consolidated or not depends directly on the timing of the curing stage and the curing speed, in which the influence of temperature on the curing speed is dominant [12]. At present, the vast majority of researchers determined the cell nucleation process through the final cell structure [13,14,15,16]. In order to further explore the law of cell formation in the foaming process, visualization technology has been used at home and abroad to track the whole process of foaming nucleation [17,18,19]. This technology will also be the trend of regulating foaming quality in the future.

Remon et al. [20] designed hot-stage optical microscope computer digital imaging visualization equipment to study the preparation of polymer foaming materials by chemical foaming method under low-pressure conditions. The results showed that the foaming process of polypropylene (PP) experienced four different stages: pre-nucleation, nucleation, cell growth, and cell shrinkage. Leung et al. [21] discussed the change in the critical nucleation radius under different gas concentrations and the influence of these changes on the cell stability during the foaming process of the low-density polyethylene (LDPE)/ Chemical Blowing Agent (CBA) system with the aid of a visualization device. Those results showed that the cell experienced two stages: cell growth and cell collapse. Kentaro et al. [22] observed the effect of nano-fillers on cell nucleation and growth in the early stage of PP foaming through a new high-pressure autoclave device, and found that nano-fillers greatly increased the probability of cell nucleation, resulting in the decrease of CO_2_ concentration in the resin matrix. Anson Wong et al. [23] designed a visualization device with reversible rotating rollers to study the effect of tensile strain and strain rate on the foaming behavior of polystyrene-talc composites by physical foaming. It was found that with increasing tensile strain and strain rate, the time required for the foaming process of cell formation and growth was shortened, and the cell density increased significantly. Park et al. [24] successfully assembled a visualization system with precise program-controlled heating/cooling, which has been used to study the influence of polymer crystal formation on cell formation and growth. It was found that with decreasing temperature, the crystals gradually formed and grew into spherulites. Because CO_2_ did not gather in the front of the crystal growth, the nucleation of the cell first appeared at the edge of the crystal. This system can be effectively applied to study the relationship between crystallization kinetics and cell nucleation, growth, and deterioration during the foaming process. Huang and Wu et al. [25,26,27] also performed a lot of work on visualization of free foaming, exploring the process of cell nucleation and growth under low pressure. By changing the experimental temperature, adding nucleating agent, selecting a different matrix, and other process parameters, it was found that there were “under foaming”, “balanced foaming”, and “over foaming” states in the process of cell nucleation and growth. In this series of investigations, researchers adopted new characterization techniques to more accurately describe the whole process of cell nucleation and growth. Because the visualization device is able to accurately obtain the number of cells at different times, it can quantitatively reflect the whole process of cell formation in real time. However, the current research has not systematically analyzed the influence of the foaming environment on the foaming process from the variation of cell number and time.

Therefore, in this paper, with the help of the hot-stage visualization device, the PP foaming materials under different process conditions were prepared. By studying the relationship between the number of cells (*n*) and the foaming time (*t*), the influence of the foaming environment on the change in cell number was analyzed theoretically, so as to realize the regulation of cell structure parameters of foaming polyolefin materials. This study provides a theoretical basis for obtaining high-quality foamed polymer composites.

## 2. Experimental Section

### 2.1. Experimental Materials

The polymer used for free foaming was a polypropylene resin (L5E89) from China National Petroleum Corporation (Beijing, China). The chemical foaming agent was azodicarbonamide (AC), with a gas output of 220 mL/g, acquired from Wuhan Hanhong Chemical Plant (Wuhan, China). Low-density polyethylene (LDPE, 2426H) was used as the matrix for foaming agent masterbatch preparation, with a density of 0.93 g/cm^3^ and a melt flow rate of 1.9 g/10 min, from PetroChina Lanzhou Petrochemical Company (Lanzhou, China). The foaming agent masterbatch was self-prepared [28].

### 2.2. Sample Preparation

PP and the foaming agent were mixed uniformly according to a certain ratio (Table 1), and unfoamed splines with different foaming agent contents were prepared by a foaming injection molding machine with a controllable cavity volume (EM120-V, Zhende Plastic Machinery Co., Ltd., Guangdong, China). The thickness of the splines was controlled within 0.65–0.7 mm, and the splines were cut into small splines of 4 × 4 mm.

**Figure 1:**
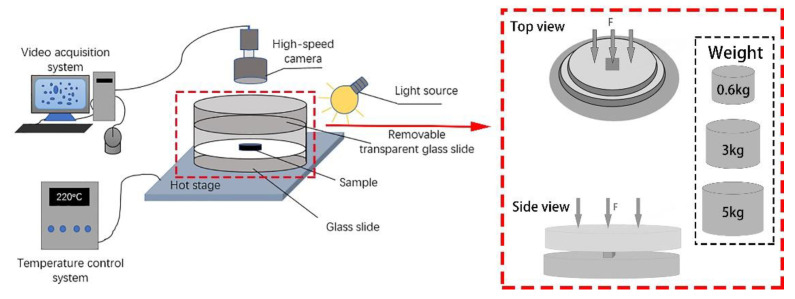
Simple optical microscope visualization device model diagram.

The prepared samples were placed on a constant temperature heating stage for melting. However, the sample will shrink when heated, which was not conducive to visual observation. Therefore, a 0.6 kg weight should be added to the up-slide to prevent melt shrinkage. When the effect of different external forces on the PP foaming process was observed, the external force changed from 0.6 to 3 kg and 5 kg. The schematic diagram is shown in Figure 1.

### 2.3. Injection Molding Experiment

**Figure 2:**
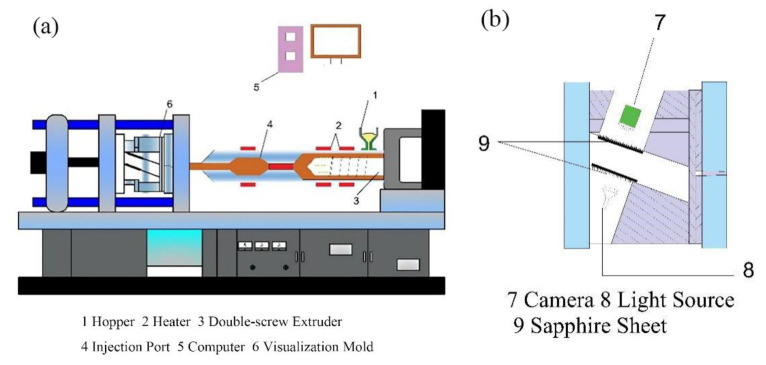
(**a**) Injection molding device diagram, (**b**) The visualization mold diagram.

The matrix PP and foaming agent masterbatch were evenly mixed in a certain proportion and added into the cavity volume controllable foaming injection molding machine (TTI-205Ge, Donghua Machinery Co., Ltd., Dongguan, China). The device diagram is shown in Figure 2 [29]. PP foaming materials were prepared by the core-back injection process and the short-shot injection process. The process conditions are listed in Table 2.

### 2.4. Visualization Device

Figure 1 is a visualization device for observing the foaming process in a free foaming environment. The equipment consists of a high-speed camera CCD system (TK-C1031EC, Guangzhou, China), image processing system, transparent visualization mold (quartz glass), hot-stage (WT-3000-12s, Shanghai, China), and light source. The high-speed camera system is connected to the image processing system, and the hot-stage has a stable constant temperature system [26]. The whole foaming process captured by the high-speed camera system is transmitted to the image processing system. Finally, the number of cells in the picture is counted by using nano-measurer software to obtain the change in the number of cells at different times.

## 3. Results and Discussion

### 3.1. Effect of Melt Temperature on Cell Formation

The foaming process of PP at different temperatures was observed continuously by the visualization equipment, and the temperature was set at 200–230 °C. The results were as follows.

**Figure 3:**
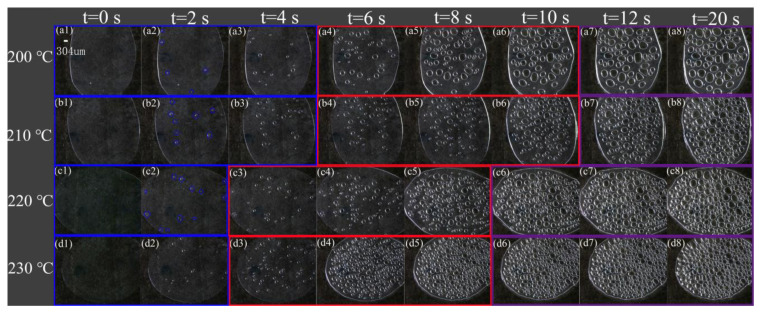
Video screenshots are at different times at different temperatures.

**Figure 4:**
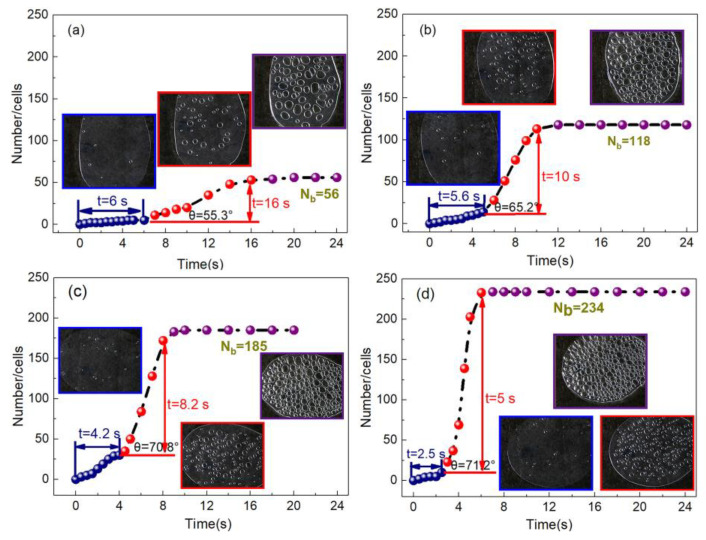
The *n*-*t* curves under different temperatures: (**a**) 200 °C, (**b**) 210 °C, (**c**) 220 °C, (**d**) 230 °C.

Figure 3 and Figure 4 are the respective visual video screenshots of the melt temperature at 200–230 °C and the *n*-*t* curves of cell number with foaming time. It can be seen that at different melt temperatures, the change in *n* with *t* presents three stages of “slow increase (blue), rapid increase (red), and basic stability (purple)”, that is, the *n*-*t* curve presents a typical “S” shape. In the slow increasing stage of the *n* value, the time is gradually shortened from 6 to 2.5 s with the increasing temperature, which indicates that the increasing foaming temperature effectively shortens the time of the cell number slowly increasing stage, and the next stage begins earlier. In the stage of rapid cell formation, with the rising temperature, the time of rapid cell formation is advanced, and the speed of cell formation is also gradually accelerated. In the stage of cell stabilization, the *n* value is stable at 200 °C after 18 s, while the stable time is shortened by 6 s at 230 °C, and the final *n* value increases from 56 to 234.

**Figure 5:**
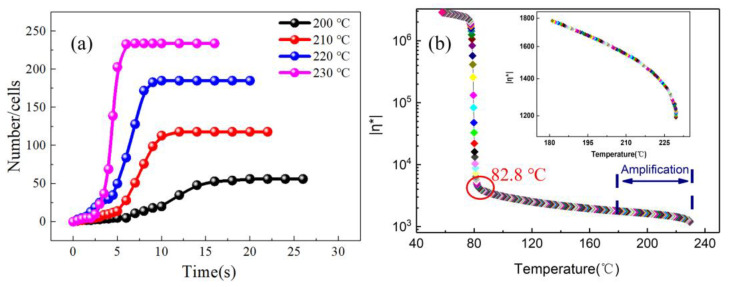
(**a**) The *n*-*t* curve comparison chart at 200–230 °C; (**b**) The curve of viscosity change with temperature.

The influence of the melt temperature on cell formation mainly lies in two aspects: firstly, formula (1) is a modified form of the logistic model to describe the relationship between cell number *n* and time *t* [29]. Through the establishment of the mathematical model, the Gibbs free energy Δ*G* at different temperatures can be fitted (Table 3). With the increased temperature, the Δ*G* values are 33.85, 29.6, 27.15, and 26.05 kJ. The decrease in Δ*G* indicates that the energy barrier of cell formation was lower, which is more conducive to cell formation. Secondly, according to Henry’s law and the critical nucleation radius formula [30] (formula (2), formula (3)), the cells will expand spontaneously only when the cell size is larger than the critical nucleation radius (*R*_C_) in formula (3); otherwise, the cells will disappear. With the increasing temperature, the melt viscosity decreases (Figure 5b), which will lead to an increase the free volume and the molecular spacing in the polymer and will weaken the interaction between molecules. Therefore, when the temperature rises, the surface tension ($\sigma_{GL}$) and the critical nucleation radius of the cells decrease, which are more beneficial to the cell nucleation, and the time of cell formation is advanced.

(1) $$N=10\times N_{1}\times \frac{\exp\left[n\times \left(t-\tau \right)\right]}{1+\exp\left[n\times \left(t-\tau \right)\right]}\times \exp\left(-\frac{\Delta G}{kT}\right)$$

(2) $$P_{bub}=H\cdot C$$

(3) $$R_{c}=\frac{2\sigma_{GL}}{\;P_{bub}-P_{sys}}$$

where *n* is the number of cells at time (*t*). The effective nucleation site *N*_1_ = 2000. *n* is the bubble nucleation index. *T* is the foaming temperature, τ is the delay time. Δ*G* is the critical nucleation Gibbs energy. *R*^2^ is the regression correlation coefficient. *C* is the solubility of gas and melt. H is Henry’s constant. $R_{c}$ is the critical nucleation radius. $\;P_{bub}$ is the pressure in the cell. $P_{sys}$ represents the system pressure, which equals the atmospheric pressure. $\sigma_{GL}$ is the surface tension between gas and melt, and $k_{H}$ is Henry’s constant.

### 3.2. Effect of Foaming Agent Content on Cell Formation

**Figure 6:**
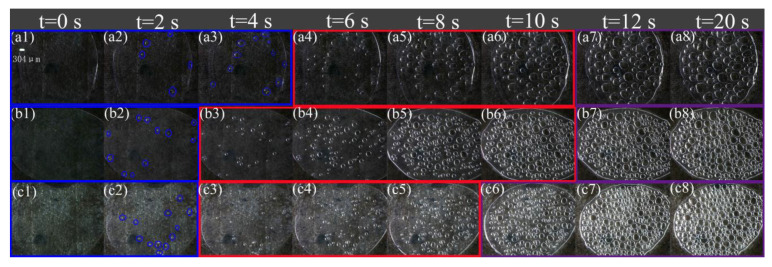
Video screenshots at different times: (**a**) 0.5 wt.%, (**b**) 0.6 wt.%, (**c**) 0.7 wt.%.

**Figure 7:**
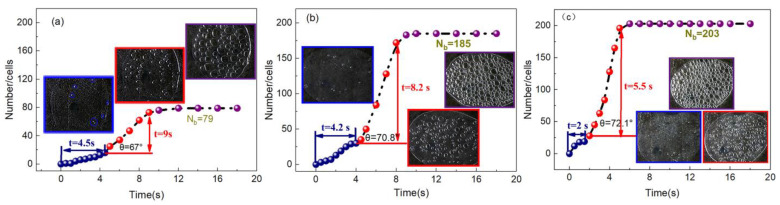
The *n*-*t* curves under different foaming agent contents: (**a**) 0.5 wt.%, (**b**) 0.6 wt.%, (**c**) 0.7 wt.%.

Figure 6 and Figure 7 are visual video screenshots and corresponding *n*-*t* curves under different foaming agent contents, respectively. It can be seen that the change in the foaming agent content does not significantly change the process of cell formation, that is, increasing cell number still includes slow growth, rapid growth, and the final stable period, and the *n*-*t* curves still maintain the typical “S” shape. When the content of foaming agent is 0.5 wt.%, the cell number slowly increases to 4.5 s, while when the content of foaming agent is 0.7 wt.%, it is shortened to 2 s, which indicates that the increasing foaming agent content can effectively shorten the time of cell formation and improve the rate of cell formation. In the stage of rapid cell formation, the angle *θ* of the *n*-*t* curve represents the cell formation rate, and the value increases with increasing foaming agent content. The higher the *θ* value, the higher the cell formation rate. In the stage of cell stabilization, the final cell number (*N*_b_) increases from 79 to 203 with increasing foaming agent content, and the time to enter the stable stage is shortened from 9 to 5.5 s.

**Figure 8:**
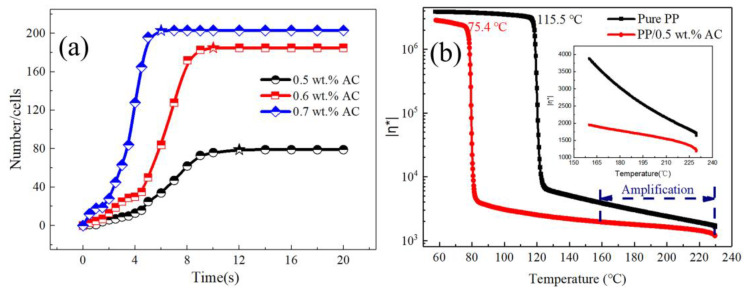
(**a**) Comparison of *n*-*t* curves with different foaming agent contents; (**b**) Curves of viscosity of pure PP and PP/AC with changing temperature.

There are two effects of the foaming agent content on cell formation: firstly, in the free foaming process, the cell formation and growth process are regarded as a constant pressure process, and $\;P_{bub}-P_{sys}$ represents the driving force of nucleation. In the case where other conditions remain the same, when the percentage of dissolved gas in the system increases, the pressure in the cell will be also enhanced, which increases the pressure difference inside and outside the cell, resulting in the decline in the critical nucleation radius (formula (3)) [31]. Secondly, when the foaming agent is added to the matrix, the gas generated by heating will disperse in the melt, which leads to the decrease of melt viscosity (Figure 8b). The relationship between $\sigma_{GL}$ and melt viscosity is a positive correlation; thus, the resistance in the process of cell formation is reduced, and the cell formation rate is increased, so more cells are formed. This is obtained by fitting formula (1); as the content of the foaming agent increases, Δ*G* decreases to 32.9, 31.25, and 31.05 kJ (Table 4).

### 3.3. Effect of Melt Pressure on Cell Formation

**Figure 9:**
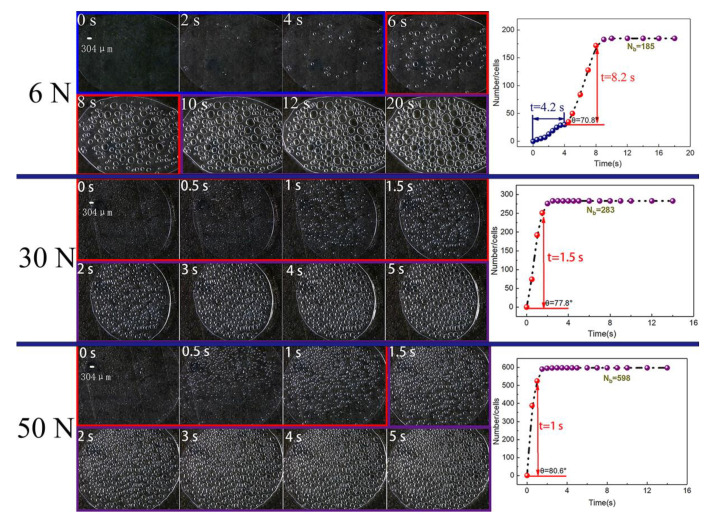
Video screenshots and the *n*-*t* curve at different times with different external forces.

**Figure 10:**
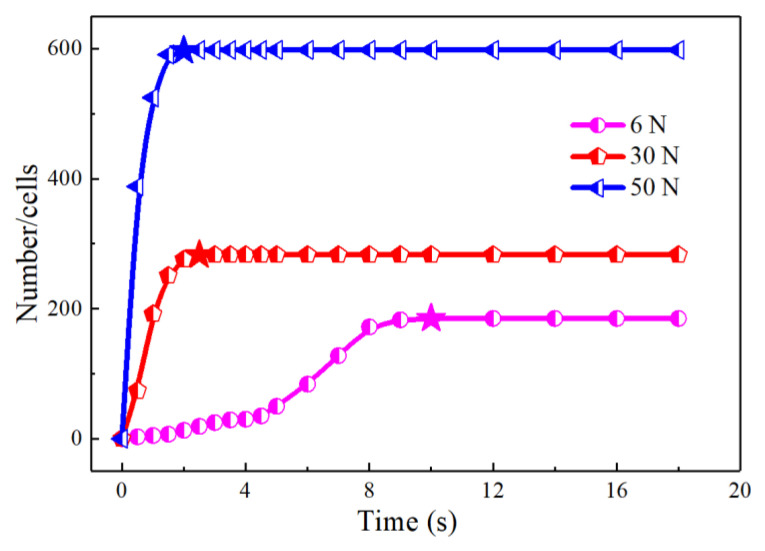
The *n*-*t* curves under different external forces.

Figure 9 and Figure 10 display the video screenshots and *n*-*t* curves under different external forces. By comparison, it was found that when the external force was 6 *n*, the cell formation process still contained the above three stages. It took 4.5 s for the cell number to slowly increase, but with the increase in external force, the stage in which slow cell formation occurred disappeared, and the shape characteristics of the *n*-*t* curve changed from a typical “S” shape to a “semi-S” shape. In addition, the rate of cell formation increased with an increase in the external force. The time for the stage of cell stabilization decreased from 8.2 to 1 s, and the maximum cell number increased from 185 to 598. A series of experimental results showed that increasing melt pressure effectively reduced the cell nucleation barrier and accelerated cell nucleation.

In the atmospheric pressure foaming process, the foaming agent in the melt is decomposed by heating and applying a certain external force. Then, when the external force is removed, the pressure of the polymer/gas single-phase system decreases rapidly. The saturated polymer/gas system gradually turns into an unsaturated system and enters into a thermodynamic unstable state. The separation of gas and PP begins to form cells to reduce the free energy of the system. The faster the PP melt decreases from a high pressure to a certain stable pressure, the greater the driving force for cell nucleation. Therefore, in this process, the magnitude of the external force has a great influence on the melt pressure, that is, the higher the applied force, the greater the pressure of the polymer melt. When the external force is removed, the pressure drop rate increases, and the system quickly reaches the thermodynamic instability state, which leads to a rapid rise in cell number and a short time for cell stabilization, thus improving the efficiency of cell formation [32,33]. According to formula (1), with the increase in melt pressure, Δ*G* decreased obviously (Table 5). This means that the increase in melt pressure effectively reduced the resistance of the cell formation process, thereby increasing the cell formation rate.

### 3.4. Effect of Melt Pressure on Cell Formation in the Actual Injection Molding Process

**Figure 11:**
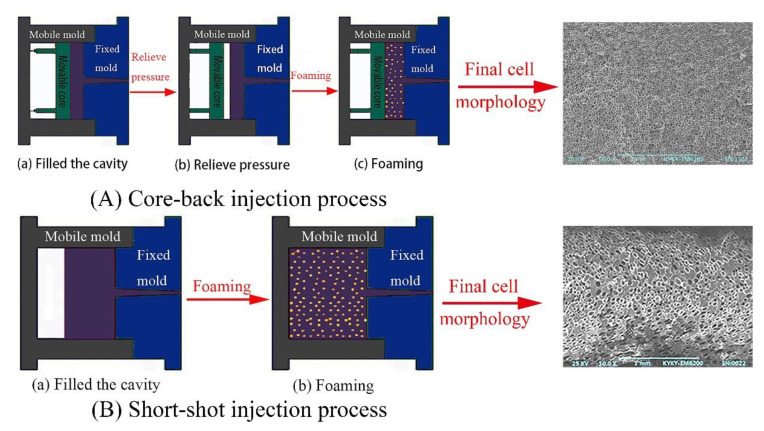
Schematic of the foam material process forming of (**A**) the core-back injection process and (**B**) the short-shot injection process.

**Figure 12:**
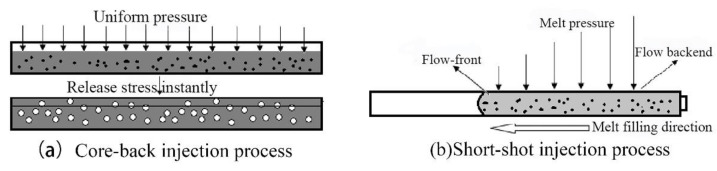
Schematic diagram of the pressure release force of melt in (**a**) the core-back injection process, (**b**) the short-shot injection process.

In the actual injection molding process, the core-back injection process (Figure 11A) and the short-shot injection process (Figure 11B) are the two most commonly used molding processes for preparing foaming materials, and the greatest difference between the two processes is the melt pressure in the cavity [4]. In the core-back injection process, the cells are in a uniform pressure environment (Figure 12a), and the short-range migration of resin is driven by the instantaneous gas expansion, and each cell is in the same pressure environment [34,35]. On the contrary, the short-shot injection process is a foaming process in which melt filling and cell growth occur simultaneously, and the pressures on the different parts of PP melt are different [36] (Figure 12b). In the front part of the melt flow, the melt has no cavity constraint in one direction, but near the end part of PP melt flow, there are constraints of the melt and cavity around the cells, where the pressure in the melt is greater than that in the front part.

In order to compare the injection molding process with the free foaming process, the influence of melt pressure on cell nucleation in the core-back injection process and the short-shot injection process were investigated by visualization means and a pressure sensor device. The specific process parameters are presented in Table 2.

**Figure 13:**
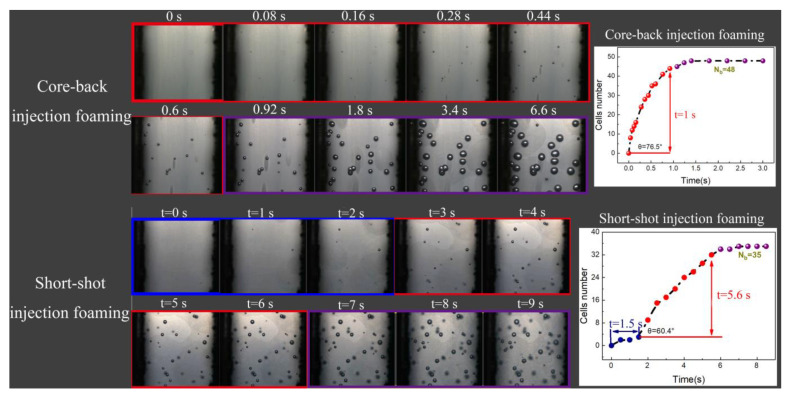
Change in cell number with time in the core-back injection process and the short-shot injection process.

Figure 13 shows the video screenshots of foaming materials prepared by the core-back injection process and the short-shot injection process, and the *n*-*t* curves were drawn by the video screenshots. It can be clearly observed that in the core-back injection process, the time to the stage of cell stabilization is only 1 s, and the maximum number of cells is 48. There are only two stages of cell nucleation in the core-back injection process, that is, the stage of rapid cell formation and the stage with a stable cell number. However, in the process of short-shot injection foaming, the time to the stage of cell stabilization is close to 7 s, and the value of *θ* is smaller than that of core-back molding, which indicates that the rate of cell formation is slower. The most obvious characteristic of the core-back injection process is that no slowly rising cell number stage can be observed, and the *n*-*t* curve changes from an “S” shape to a “semi-S” shape.

**Figure 14:**
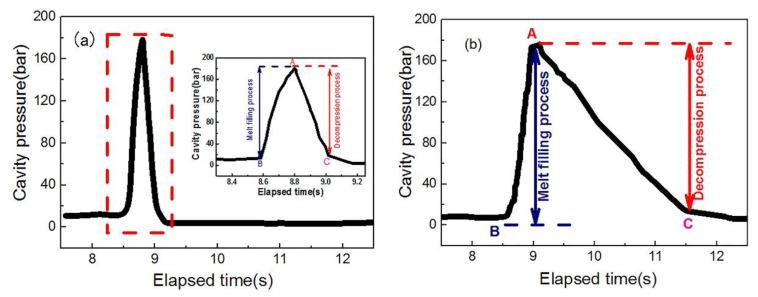
Diagram of the cavity pressure change in (**a**) the core-back injection process and (**b**) the short-shot injection process.

As shown in Figure 14a, it was found that from point B to point A in the core-back injection process, a melt filling process takes place, and the cavity pressure increases rapidly at this stage until the completion of filling at point A. The cavity pressure reaches the maximum value of 180.02 bar. On the contrary, for the short-shot injection process shown in Figure 14b, the process of pressure reduction from point A to point C is 2.51 s, which is about ten times that of the core-back injection process, indicating that the slower pressure drop rate cannot provide enough driving force for the formation of cells, which reduces the number of cells and slows down the cell growth.

## 4. Conclusions

(1) In the free foaming environment, the change in *n* with *t* presents three stages: slow increase, rapid increase, and basic stability, irrespective of whether the melt temperature and contents of the foaming agent changed. With the increase in temperature and the foaming agent content, the time of the slow growth stage of the cell number was gradually shortened, and the latter two stages were advanced.
(2) The melt pressure had a significant effect on the process of cell formation. With increasing melt pressure, the *n*-*t* curve changed from a complete “S” shape to a “semi-S” shape. This change presented two stages of “rapid increase and basic stability”, the absence of a slow increase in cell number, and the rate of cell formation increased significantly. With increasing melt pressure, the cell can form in advance.
(3) In the actual dynamic foaming process of injection molding, under the condition of a short-shot injection process, the *n*-*t* curve exhibited a typical “S” shape. Under the condition of the core-back injection process, the *n*-*t* curve changed from the complete “S” shape to the “semi-S” shape. This meant the cell formation presented only two stages; there was the absence of a slow formation stage, and the formation rate of new cells increased significantly. The cell formation laws of the actual injection dynamic foaming process were consistent with the influential law in the free foaming environment.

## Figures and Tables

**Table 1:** The proportion table of PP composite foam material.

Component	PP	Foaming Agent Masterbatch
1	99.5 wt.%	0.5 wt.%
2	99.4 wt.%	0.6 wt.%
3	99.3 wt.%	0.7 wt.%

**Table 2:** Process parameters in core-back injection and short-shot injection.

Molding Process	Melt Temperature	Injection Speed	Injection Pressure	Injection Volume	Mold Opening Distance	AC Content
Core-back injection	200 °C	30%	40 bars	22 mm	0.6 mm	0.6 wt.%
Short-shot injection	200 °C	30%	40 bars	22 mm	----	0.6 wt.%

**Table 3:** Influence of melt temperature on the parameters.

Melt Temperature	*N*_1_	*τ*	*n*	*T*	Δ*G*	*a*	*R* ^2^
200 °C	2000	10.3	0.42	473	38.35	0	0.9945
210 °C	2000	7.2	0.75	483	34.2	0	0.9961
220 °C	2000	6.4	0.9	493	31.25	0	0.9921
230 °C	2000	4.3	1.9	503	30.9	0	0.9963

**Table 4:** Influence of the content of the foaming agent on the parameters.

Content of Foaming Agent	*N*_1_	*τ*	*n*	*T*	Δ*G*	*a*	*R* ^2^
0.5 wt.%	2000	6.6	0.7	493	32.9	0	0.9970
0.6 wt.%	2000	6.4	0.9	493	31.25	0	0.9921
0.7 wt.%	2000	3.5	1.4	493	31.25	0	0.9954

**Table 5:** Influence of the melt pressure on the parameters.

Melt Pressure	*N*_1_	*τ* (s)	*n*	*T* (K)	*G* (kJ)	*a*	*R* ^2^
6N	2000	1.8	2.8	493	32.9	0	0.9897
30N	2000	0.7	4.4	493	28.7	0.006	0.9890
50N	2000	0.5	6.5	493	23.65	0.01	0.9703

## Data Availability

Not applicable.
